# Cell therapy for cartilage repair

**DOI:** 10.1042/ETLS20210015

**Published:** 2021-08-23

**Authors:** Charlotte H. Hulme, Jade Perry, Helen S. McCarthy, Karina T. Wright, Martyn Snow, Claire Mennan, Sally Roberts

**Affiliations:** 1 School of Pharmacy and Bioengineering, Keele University, Keele, Staffordshire, U.K.; 2 Robert Jones and Agnes Hunt Orthopaedic Hospital, Oswestry, Shropshire, U.K.; 3 The Royal Orthopaedic Hospital, Birmingham, U.K.

**Keywords:** cartilage, cell therapy, chondrocytes, mesenchymal stem cell, osteoarthritis, regenerative medicine

## Abstract

Regenerative medicine, using cells as therapeutic agents for the repair or regeneration of tissues and organs, offers great hope for the future of medicine. Cell therapy for treating defects in articular cartilage has been an exemplar of translating this technology to the clinic, but it is not without its challenges. These include applying regulations, which were designed for pharmaceutical agents, to living cells. In addition, using autologous cells as the therapeutic agent brings additional costs and logistical challenges compared with using allogeneic cells. The main cell types used in treating chondral or osteochondral defects in joints to date are chondrocytes and mesenchymal stromal cells derived from various sources such as bone marrow, adipose tissue or umbilical cord. This review discusses some of their biology and pre-clinical studies before describing the most pertinent clinical trials in this area.

## Introduction

Articular cartilage is a specialised connective tissue within synovial joints, providing a smooth lubricated surface to reduce friction and resist compressive forces during movement. It is aneural and avascular, with an extracellular matrix (ECM) comprised of an intricate network of collagen fibres, proteoglycans and non-collagenous proteins. Articular cartilage is renowned for its inability to repair itself once damaged [1] and, if left untreated, often progresses into osteoarthritis (OA) [2]. OA is very prevalent in the knee and hip [3] and results in significant pain and disability for the patient, with limited treatment options, until reaching end-stage disease, when joint replacement is likely. The ensuing economic burden has resulted in OA being described as a Serious Disease by an Osteoarthritis Research Society International (OARSI) White Paper [4]. Currently, there are approximately 200 000 hip and knee replacement operations each year in the U.K. (except Scotland), with OA listed as the clinical indication in over 90% of cases [3]. This has increased ∼10% annually in the last decade due to demographic and obesity changes [3]. Thus, the endeavour for an effective treatment to halt, or even reverse the progression of OA is ever more pressing.

Current surgical interventions to treat focal chondral or osteochondral lesions include microfracture, osteotomy, mosaicplasty and osteochondral autograft transplantation (OATS), each with their own advantages and disadvantages. Microfracture, a type of bone-marrow stimulation, is commonly used for the treatment of small (<2 cm^2^) chondral lesions but is not suitable for larger defects or those that extend into the subchondral bone [5]. Mosaicplasty traditionally requires the implantation of multiple small osteochondral grafts into the recipient defect whereas OATS involves the transfer of larger osteochondral grafts (up to 10 mm diameter) [6,7]. These techniques both result in the immediate replacement of hyaline cartilage and restoration of the articulating surface, although donor-site morbidity is a potential complication [8,9]. Interest in a more biological approach as an alternative to these surgeries, through tissue engineering to regenerate hyaline cartilage, has grown tremendously over the past two decades. This review will discuss the history, development and current concepts of different approaches for cartilage repair.

## Cell therapies in the clinic for cartilage repair

**Figure 1.**
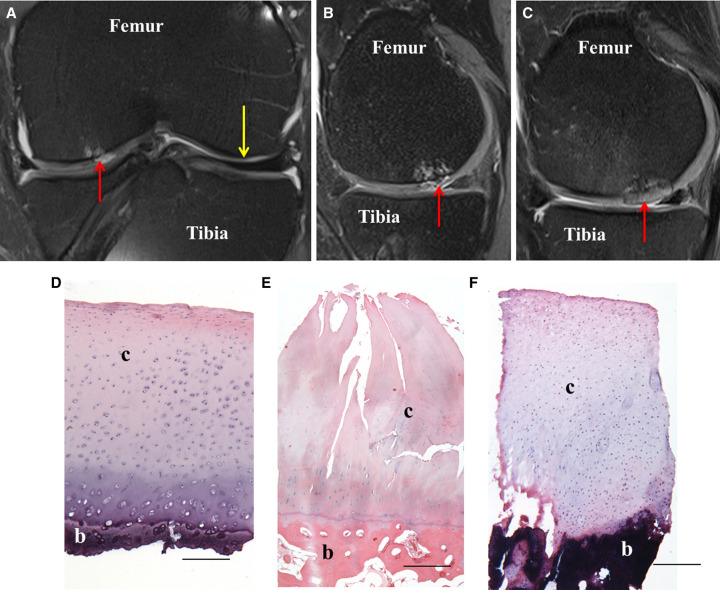
Cell therapy repair of cartilage defects. (**A**–**C**) Representative 3-T magnetic resonance imaging (MRI) scans from a 35-year-old patient who underwent treatment with ACI. Proton density-weighted turbo spin-echo fat-suppressed (PD-TSE-FS) sequence MRI, showing (**A**) coronal and (**B**) sagittal view of the knee joint with an osteochondral defect (red arrow) prior to ACI treatment and (**C**) the treated defect 13 months post-ACI. Yellow arrow indicates normal healthy cartilage. D-F) Representative histological images of haematoxylin and eosin-stained sections of (**D**) normal, healthy articular cartilage, (**E**) fibrillated, degenerative cartilage and (**F**) repair tissue formed 12 months post-ACI. c, cartilage; b, bone. Scale bars = 500 µm.

The repair of cartilage defects has been at the forefront of regenerative medicine applications. Autologous Chondrocyte Implantation (ACI) is one such approach which has been used in the clinic for almost 30 years (Figure 1) [10]. Although ACI has evolved over time, the premise of this technique is to arthroscopically harvest cartilage from a low-weight bearing region of the joint. This cartilage is enzymatically digested to release chondrocytes, which are then culture-expanded prior to being implanted into the chondral or osteochondral defect under a native or synthetic membrane in an open arthrotomy procedure [11]. In its first iteration, ACI was performed using a periosteal patch, harvested from the tibia, under which the expanded chondrocytes were injected. Complications relating to graft hypertrophy [12], graft failure [13] and difficulties in suturing the periosteal patch [14] resulted in the development of 2nd generation ACI, ACI using a porcine collagen types I and III-containing membrane (Chondro-Gide^TM^, Geistlich, Switzerland), as an alternative patch. Within 10 years of ACI first having been reported, further modifications resulted in the development of 3rd generation ACI: matrix-autologous chondrocyte implantation (MACI^TM^)[15], which consists of culture-expanded chondrocytes that are suspended in a hydrated scaffold (Vericel, U.S.A.), without the need for any membrane or patch [15]. Overall, the long-term evidence for ACI is very encouraging and when considered in terms of the cost per quality-adjusted life-year (QALY), sufficient evidence could be drawn to result in it being recommended by the UK National Institute for Health and Care Excellence (NICE) for the treatment of cartilage lesions greater than 2 cm^2^ [16,17].

More recently a potential 4th generation of ACI has been developed, with the aim to better replicate the innate cartilage prior to implantation. Spheroids of neocartilage comprising of expanded autologous chondrocytes with their associated pericellular matrix are implanted without the need for a synthetic matrix, facilitating arthroscopic implantation [18,19]. Manufactured by CO.DON AG, Spherox or Chondrosphere^TM^ is currently the only commercially available ACI approved by NICE [20].

ACI (including MACI^TM^) is the preferred option for the treatment of chondral/osteochondral defects greater than 2 cm^2^, particularly if those patients who are likely to benefit can be selected for treatment [21,22]. However, it is not without its limitations. Primarily, regulatory constraints restrict the number of hospitals that can currently provide this surgical option within the U.K. The culture expansion of chondrocytes for clinical use is expensive, highly regulated and requires a good manufacturing practice (GMP) facility to be able to receive and culture-expand the cells for a period of 3–6 weeks. In the U.K., manufacturers would require licenses from both the Human Tissue Authority and Medicines and Healthcare products Regulatory Authority (MHRA). Currently, there are no U.K. based commercial manufacturers of expanded chondrocytes, with only Co.Don (from Berlin) operating here, providing Chondrosphere™. The challenging commercial environment has consequently resulted in many entities withdrawing from the market, e.g. Carticel^TM^ (Genzyme, U.S.A.) [23] and Chondroselect (Tigenix, Belgium). The regulation and governance of cell therapy treatments (constituting an advanced therapeutic medicinal product (ATMP)) are greater than for other pharmaceutical products and can be considered a burden for both research and development into a commercial product [24]. Moreover, running clinical trials with ATMPs often poses specific challenges to their design and conduct [25]. In addition, when using an autologous product such as in ACI, the need for two surgical procedures is costly and logistically challenging. The two-stage process also exposes the patient to increased clinical risk and requires a long period of post-surgical rehabilitation [26]. Other considerations include donor-site morbidity at the site of the cartilage harvest [27], although some authors suggest that this can heal naturally [28] with no morbidity after 12 months if taken from the medial or central trochlea [29]. Although ACI has been demonstrated as a viable option in terms of cost-benefit analysis [17,20], a single-stage allogeneic cell therapy with large-scale cell manufacture from donors potentially has both clinical and economic advantages.

## Clinical trials of chondrocytes

The level I and II clinical evidence for ACI in the treatment of cartilage lesions in the knee is summarised in Table 1. The SUperiority of Matrix-induced autologous chondrocyte implantation versus MIcrofracture for Treatment of symptomatic articular cartilage defects (SUMMIT) trial (http://clinicaltrials.gov: NCT00719576), compared MACI^TM^ against microfracture. Superior improvements were found in the Knee injury and Osteoarthritis Outcome Score (KOOS) for the MACI group at 5-year follow-up [30]. The TIG/ACT (TiGenix) trial (http://clinicaltrials.gov: NTC00414700) compared ACI using characterised chondrocytes against microfracture and showed improvements for both techniques in terms of KOOS up to 60 months post-op. Further subgroup analysis revealed that ACI treated participants had superior KOOS when treatment was undertaken within 3 years of symptom onset [31]. The autologous chondrocyte transplantation/implantation versus existing treatments (ACTIVE) trial (https://www.isrctn.com: ISRCTN48911177) compares ACI against surgeon selected standard of care, the majority being microfracture. Data from this ongoing trial contributed to the positive NICE Health Technology Assessment (HTA) TA477 of ACI in 2017 but the results from the study cannot yet be published until trial completion. Based principally on functional outcome over time and survival analysis the concluding message from the HTA was that ACI offered long-term superiority compared with microfracture and was ‘cost-effective across a range of scenarios’ [17].

**Table 1.** Clinical trials using chondrocytes to repair chondral defects, registered on Clinical Trials.gov or the International Standard Randomised Controlled Trial Number (ISRCT) databases

Trial name	Trial ID (Clinicaltrials.gov or ISRCTN.com)	Trial summary	Study participants	Participant age	Location(s)	Related publications
SUMMIT — Superiority of matrix-induced autologous chondrocyte implant versus microfracture for treatment of symptomatic articular cartilage defects	NCT00719576	Autologous cultured chondrocytes on porcine collagen membrane (MACI) vs. microfracture	144	18–55	Czechia, France, Netherlands, Norway, Poland, Sweden, U.K.	[30]
TIGACT01 — RCT of ChondroCelect® (in an ACI procedure) vs. microfracture in the repair of cartilage defects of the knee	NCTC00414700	ChondroCelect® implantation procedure (ACI) vs. microfracture	118	18–50	Belgium, Croatia, Germany, Netherlands	[93]
An investigational clinical trial for the safety and efficacy evaluation of Chondron^TM^ (autologous cultured chondrocyte) compared with microfracture surgery in subjects with cartilage defects of the knee joint	NCT02524509	Chondron^TM^ (gel type ACI) vs. microfracture	50	Any	Korea	[94]
An investigator clinical trial to observe effects of CHONDRON (autologous chondrocytes) for 12 months in patients with ankle cartilage defect	NCT01056900	Chondron^TM^ (gel type ACI) in the ankle	127	15–65	Korea	[95]
CS-ACI — Safety and efficacy study of cells sheet-autologous chondrocyte implantation to treat articular cartilage defects	NCT01694823	Culture chondrocyte sheets-phase I and II trial	10	18–50	China	N/A
Study to assess the safety of treatment of articular cartilage lesions with CartiLife®	NCT03545269	Bead type autologous chondrocytes vs. microfracture	30	19–65	South Korea	[96]
Phase 3 study comparison of autologous chondrocyte implantation versus mosaicoplasty	NCT00560664	ACI vs. mosaicoplasty	58	18–50	France	N/A
ASCROD — Autologous mesenchymal stem cells vs. chondrocytes for the repair of chondral knee defects	NCT01399749	ACI vs. autologous adipose MSCs	30	18–55	Spain	N/A
ACTIVE — Autologous chondrocyte transplantation/implantation versus existing treatments: a randomised controlled trial (*On-going- proposed end date Dec 2021*)	ISRCTN48911177	ACI vs. existing surgical treatments for patients who have failed a primary intervention for chondral defects	390	18+	Norway, U.K.	N/A
Introduction of ACI for cartilage repair (*on-going- proposed end date Sept 2025*)	NCT04296487	Autologous chondrocyte injection vs. standard ACI	100 (proposed number)	15–50	Switzerland	N/A
NOVOCART 3D treatment following microfracture failure (*on-going- proposed end date Dec 2021*)	NCT03219307	Safety and efficacy of matrix associated ACI (NOVOCART 3D) following microfracture	30 (proposed number)	18–66	United States	[97]
A prospective randomized controlled multicenter phase III clinical study to evaluate the safety and effectiveness of NOVOCART® 3D plus compared with the standard procedure microfracture in the treatment of articular cartilage defects of the knee (*on-going- proposed end date May 2022*)	NCT01656902	NOVOCART® 3D plus vs. microfracture	263 (proposed number)	14–65	Austria, Czechia, France, Germany, Hungary, Latvia, Lithuania, Poland, Switzerland, U.K.	[97]
A multi-center, active-controlled, ppen-label, phase 2 trial to compare the efficacy and safety of CartiLife®, and microfracture for patients with articular cartilage defects in the knee (*on-going- proposed end date Dec 2023*)	NCT04744402	ACI (CartiLife®) vs. microfracture	50 (proposed number)	19+	United States	N/A
PEAK — A study of MACI in patients aged 10–17 years with symptomatic chondral or osteochondral defects of the knee (*on-going-proposed end date June 2025*)	NCT03588975	MACI vs. microfracture	45 (proposed number)	10–17	United States	N/A
ASCOT — Autologous atem cells, chondrocytes or the two? (*On-going-proposed end date 2023*)	ISRCTN98997175	ACI vs. autologous BM-MSCs vs. combined ACI and autologous BM-MSCs	114 (proposed number)	18–80	U.K.	[46]

ACI, autologous chondrocyte implantation; BM-MSCs, bone marrow-derived mesenchymal stromal cells; MACI, matrix assisted chondrocyte implantation; RCT, randomised control trial; 3D, three-dimensional; N/A, not available.

A 2018 systematic review of five ACI trials with a mean 7-year follow-up, showed that the majority of outcome measures reported following ACI are comparable to microfracture [5]. However, in one study the Tegner patient-reported activity score improved to a significantly greater extent in the ACI group compared with the microfracture group [32]. The benefits of ACI become more apparent with longer follow-up. In a long-term study (mean 12.8-year follow-up) that retrospectively gathered data from 224 patients, 92% of participants were satisfied and would have ACI again. In addition, scores for Lysholm, TegnerWallgren and Brittberg–Peterson assessments were improved compared with preoperative levels, confirming that ACI had maintained long-term improvements. However, participants with bipolar lesions (also known as ‘kissing’ tibiofemoral lesions, i.e. opposing lesions of the tibial plateau and femoral condyle) had poorer final outcomes compared with those with multiple unipolar lesions. In addition, a 20-year follow-up study found that 79% of the 23 patients followed to 20 years were satisfied when evaluated and had not undergone arthroplasty [12].

Clinical trials involving both autologous and allogeneic chondrocytes for the treatment of chondral lesions continue. ACTIVE is a multicentre trial collecting 10-year follow-up data from 390 patients treated with ACI versus other standard treatments as its primary outcome and is due to complete at the end of 2021. The on-going Nose2Knee Phase 2 trial is examining the efficacy of autologous chondrocytes isolated from nasal septa which are expanded *in vitro* before seeding onto a collagen I/III scaffold and cultured in chondrogenic conditions to produce a mature, hyaline-like cartilage graft that is then implanted into the knee cartilage defect (http://clinicaltrials.gov: NCT02673905). Results from this trial of 108 patients are due in 2022. The INVOSSA trial is noteworthy in using both allogeneic and genetically modified chondrocytes which are currently being studied in a Phase III trial in the US for the treatment of degenerative OA in the knee (http://clinicaltrials.gov: NCT03203330). Half of the allogeneic chondrocytes, sourced from juvenile polydactyl donors, are manipulated to overexpress TGFβ-1 with the product (Invossa™ (TissueGene C)) being manufactured by the South Korean company, Kolon. The INVOSSA Phase III placebo-controlled study aims to include 510 patients, but was halted temporarily by the FDA in 2019 due to a reported cellular contaminant of the product and is currently ‘active but not recruiting’ according to the US clinical trials registry.

## ‘Stem’ cells for cartilage repair

Cells that have the potential to differentiate into any cell type are known as stem cells. Stem cells can be either embyronic, foetal or adult in origin, each having different differentiation potency [33]. Zygotic cells (prior to epiblast formation; human embryonic day 4) are the only source of totipotent cells; these can differentiate into the three primary germ cell layers, as well as, extra-embryonic tissues, e.g. the placenta. Embryonic stem cells (eSCs) and induced pluripotent stem cells (iPSCs) [34] are pluripotent and can give rise to any cells in an organism except the extra-embryonic cells. The vast majority of work aimed at using stem cells for cartilage repair, however, has focused upon multipotent stem cells, namely mesenchymal stromal cells (MSCs) that can differentiate into several closely related lineages.

**Figure 2.**
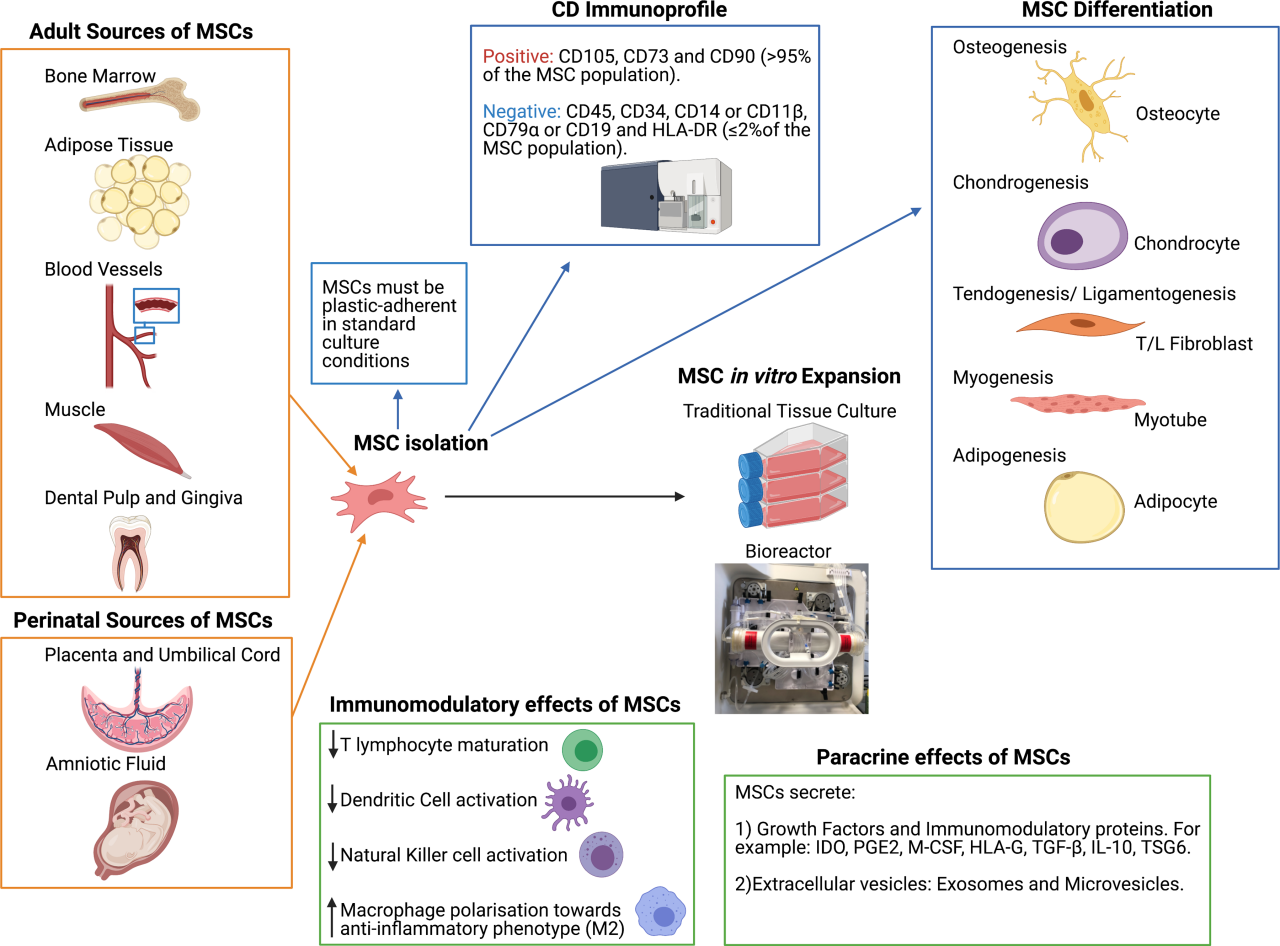
Sources and characteristics of multipotent mesenchymal stromal cells (MSCs). MSCs can be readily isolated from numerous adult and perinatal sources. The minimal criteria for MSC characterisation, published by the ISCT [35], states that MSCs must be plastic-adherent in the standard tissue culture conditions, demonstrate a specific CD immunoprofile as measure by flow cytometry (subsequently amended for adipose-derived MSCs [45]) and demonstrate a specific *in vitro* differentiation potential by differentiating down, osteogenic, adipogenic and chondrogenic lineages *in vitro* [35]*.* Specific stimuli can also promote MSCs to differentiate down myogenic and tenogenic lineages. Alongside traditional tissue culture for cell expansion, MSCs have been effectively up-scaled using bioreactors, thus enabling a switch from autologous to allogeneic multi-dose cell banking for therapeutic uses [59]. Evidence suggests that MSCs secrete large numbers of soluble and vesicle-bound growth factors and immunomodulatory proteins, which may not only have trophic effects on endogenous cells but also modulate the environment for repair [37]. (Created using Biorender.com.).

MSCs are heterogeneous cells capable of self renewal and can differentiate into various musculoskeletal lineages (osteogenic, chondrogenic and adipogenic) as well as neural precursors [35,36] (Figure 2); this heterogenous population decreases its clonal complexity with increased passaging in culture (reseeding at a lower density for the purpose of increasing cell number) [37]. It is well known, however, that BM-derived MSCs demonstrate significant heterogeneity in multi-lineage differentiation potential and clonogenicity [38]. MSCs were first identified in 1966 by Friedenstein and Petrakova, who isolated progenitor cells from rat bone marrow [39]. They were officially coined Mesenchymal ‘Stem’ Cells in 1991 [40], although this term is somewhat controversial [38], with many investigators preferring the term Mesenchymal ‘Stromal’ Cell, as the main mode of action of MSCs in regenerative medicine may not be due to their differentiation into other cell types but rather via their paracrine effects. Partly because of this, alternative names have arisen over the years such as medicinal signalling cells, mesodermal stem cells or multipotent stem cells [41]. An agreed change of name would be beneficial to the field; avoiding the use of the term ‘stem cell’ could perhaps be advantageous and reduce the hype that these cells can ‘cure’ all ailments. Clearly delineating this cell population from the ‘minimally manipulated cells’ obtained from marrow or fat may supress the recent upsurge in inappropriate commercialisation of adult MSCs and ‘stem cell tourism’, often in unregulated clinics [42].

**Figure 3.**
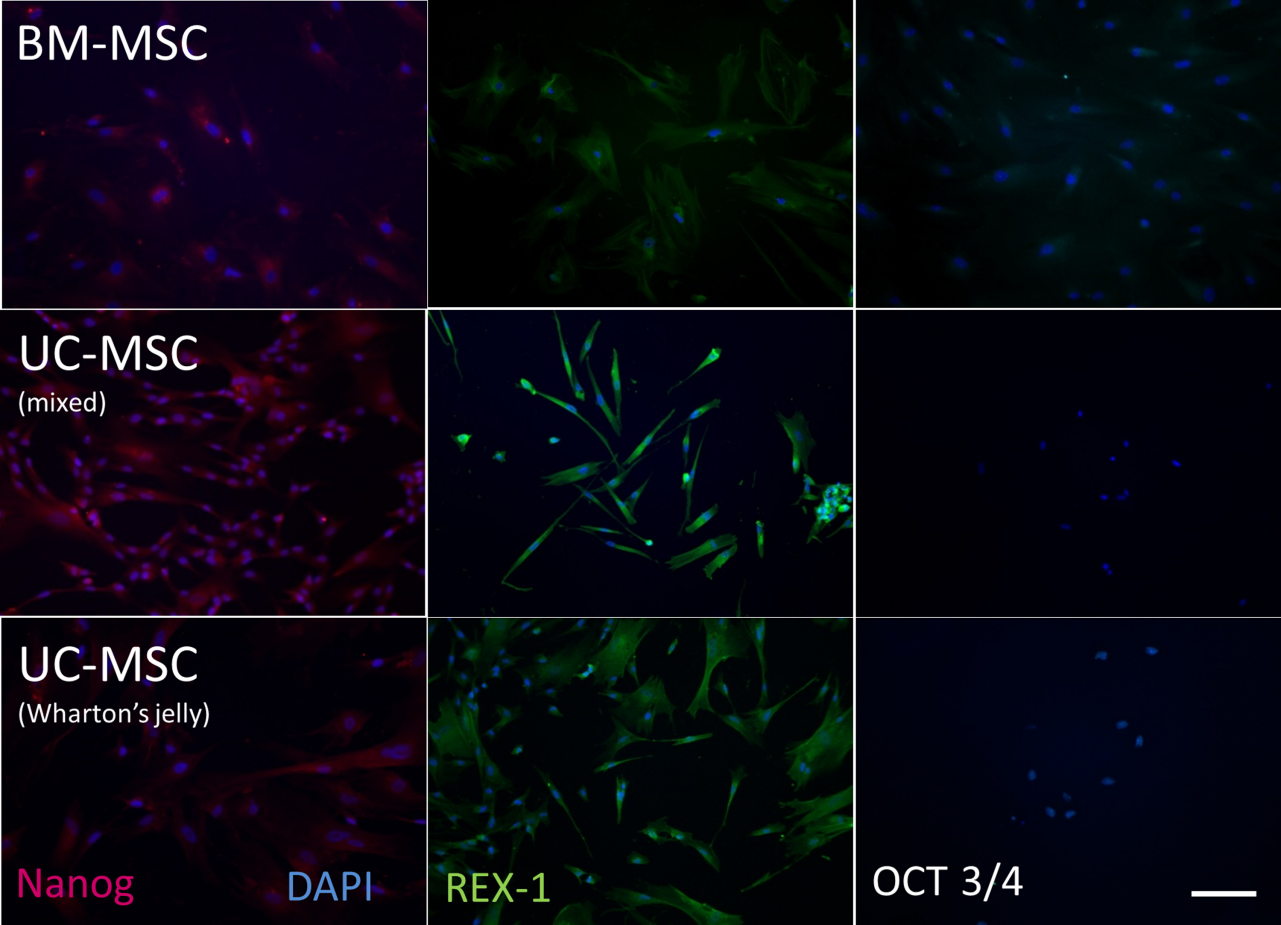
MSC expression of pluripotency markers. The expression of pluripotency markers, Nanog, REX-1 and OCT 3/4, is more common on MSCs isolated from umbilical cords (either as a mixed population from all the whole cord (mixed) or from the Wharton's jelly) than those isolated from bone marrow (BM-MSCs). Scale bar represents 100 μm. (Reproduced from [43]).

Whilst most work has focussed on MSCs isolated from bone marrow (BM-MSCs), they are increasingly sourced from other tissues such as adipose tissue, umbilical cord or placenta, with an endogenous population of progenitor cells identified in most if not all mesenchymal tissues. MSCs derived from the umbilical cord (UC-MSCs) and placenta exhibit some of the properties of eSCs as well as sharing characteristics with BM-MSC derived from adult tissue [43]. For example, unlike BM-MSC, eSCs do not up-regulate the major histocompatibility complex (MHC) class II and human leukocyte antigen-DR isotype (HLA-DR) molecules after stimulation with IFN-c [44]. eSCs are also well characterized as being pluripotent cells, that lack stage-specific embryonic antigen (SSEA)-1 but do produce SSEA-4, alkaline phosphatase, tumour repressor antigen (TRA)-1-60, TRA-1-81, OCT3/4, nanog and REX-1 (Figure 3). Unfortunately, no unique set of markers has to date been identified for MSCs *per se*. Due to the disparity of markers used by different groups, in 2006 the International Society for Cellular Therapy (ISCT) outlined the minimal criteria required to define and characterise human multipotent MSCs for use in scientific research [35] (Figure 2), which was subsequently amended for adipose-derived MSCs [45]. Clinically, BM-MSCs have been used in both autologous [46,47] and allogeneic cell therapies [48,49], whilst UC-MSCs are only proposed as an allogeneic option [50,51].

## What are the mechanisms for MSCs in cartilage repair?

For many years all ‘stem’ cells were considered useful in regenerative medicine due to their potential to differentiate into various cell types (Figure 2). The therapeutic potential of MSCs directed to differentiate into articular chondrocytes has been considered, however, there are indications that the epigenetic landscape of these cells may be distinct from matched autologous cartilage tissue [52]. However, the exact mode of action of MSCs remains to be conﬁrmed. Indeed, there is an increasing body of evidence that MSCs function through trophic effects on endogenous cells, with their production of many growth factors and cytokines, as well as by secretion of powerful immunomodulatory and anti-inflammatory molecules and extracellular vesicles (EVs) [53]. They appear to minimise any local inflammatory response in an injured or early osteoarthritic joint through the prevention of T-lymphocyte maturation, as well as the reduction in macrophage activation and secretion of immunomodulatory factors [54]. Important immunomodulatory factors produced by MSCs include indoleamine 2,3-dioxygenase (IDO), prostaglandin E2 (PGE2), macrophage colony-stimulating factor (M-CSF), human leukocyte antigen-G (HLA-G), transforming growth factor-β (TGF-β), IL-10, and tumour necrosis factor-inducible gene 6 protein (TSG6), all of which have been shown to have a role in reducing inﬂammation [55,56].

With regard to joint injury and OA, *in vivo* models suggest MSCs have been shown to have a regenerative effect in joint disease. Examples include a strong and sustained repair when autologous culture-expanded MSCs were delivered via intra-articular injection into an OA knee in a caprine medial meniscectomy (MM) model compared with no cell controls [57]. This and later studies show that these cells typically disappear from the joint within a few days and, in the goat study, any that did remain (<3%), were associated with the synovium, fat pad and meniscus [57]. Similarly, in a murine partial (P)MM model using human UC-MSCs, no cells were present in the repair tissues or indeed in the injected joint at 8 weeks post injection [58]. These experiments support the premise that MSCs did not act as cell replacement therapy but exerted their effects by an alternate paracrine mechanism.

## Large scale expansion of cells for cartilage repair

As stated, one of the major potential advantages of using MSCs (or chondrocytes) for cartilage repair is that it is possible to culture these cells on a large scale and without altering their phenotypes [59], enabling the treatment of many patients from a single batch of cells in a more cost-effective manner. Moreover, as tissue-engineering approaches aim to better recapitulate the complexity of a normal joint, the use of bioreactors can overcome the limitations of static culture environments [60]. Bioreactors can be used to mimic mechanical stimulation, hydrostatic pressure, shear and compressive forces, as well as, physiological related factors such as pH and oxygen, all of which are more permissive to chondrogenesis [61].

## Non-culture-expanded BM-MSCs

*In vitro* culture expansion of BM-MSCs can be a time consuming and costly procedure, with a significant associated regulatory burden. The use of aspirated bone marrow (bone-marrow aspirate, BMA) directly, thus negates the need for culture expansion and has generated significant interest as an alternative, low cost and easily accessible cell source (sometimes known as ‘minimally manipulated’ cells). However, of all the nucleated cells found within BMA, only ∼0.001–0.01% of these are actually MSCs [36]. This percentage can be increased by centrifugation of the aspirate, producing ‘bone-marrow aspirate concentrate’ (BMAC) which is also believed to be rich in growth factors and cytokines [62,63]. Thus autologous BMAC can be prepared in the operating theatre and implanted into a chondral or osteochondral defect in a single-stage procedure, often in combination with a scaffold, e.g. hyaluronan, fibrin gel or collagen membrane [64–67]. A recent systematic review [68] of animal and clinical studies have shown some promising early to mid-term results, although the therapy currently lacks standardisation and there is little understanding of its mechanism of action [69]. A review of the effectiveness of BMAC as an injection to treat knee OA was performed by Cavallo et al. [70]. A total of 4626 patients in 18 clinical studies were analysed and it was concluded that BMAC injections were safe and that overall improvements in pain and function were documented in most of the studies, but the overall quality of evidence was considered low.

Unfortunately, there is not a standard nomenclature accepted in the field for describing different cell populations, whether they are culture-expanded (and so more homogenous than, e.g. BMA or BMAC) nor for what characterisation has been performed, etc. The lack of a unified approach about how cell products should be described and even about their biological properties makes it easier for businesses to sell treatments allegedly based on MSCs and particularly ‘stem cells’ [42]. An international consensus group have provided good guidance on how cells applied in cell therapies should be described; the DOSES cell therapy communication tool requires reporting of five core items: donor (i.e. autologous, allogeneic, xenogeneic), origin of tissue, separation from other cell types/preparation method, exhibited cell characteristics associated with behaviour, and the site of delivery [71]. If this form of reporting is made mandatory it would clear up much ambiguity in the field.

## Pre-clinical models used for studying chondral/osteochondral repair

**Figure 4.**
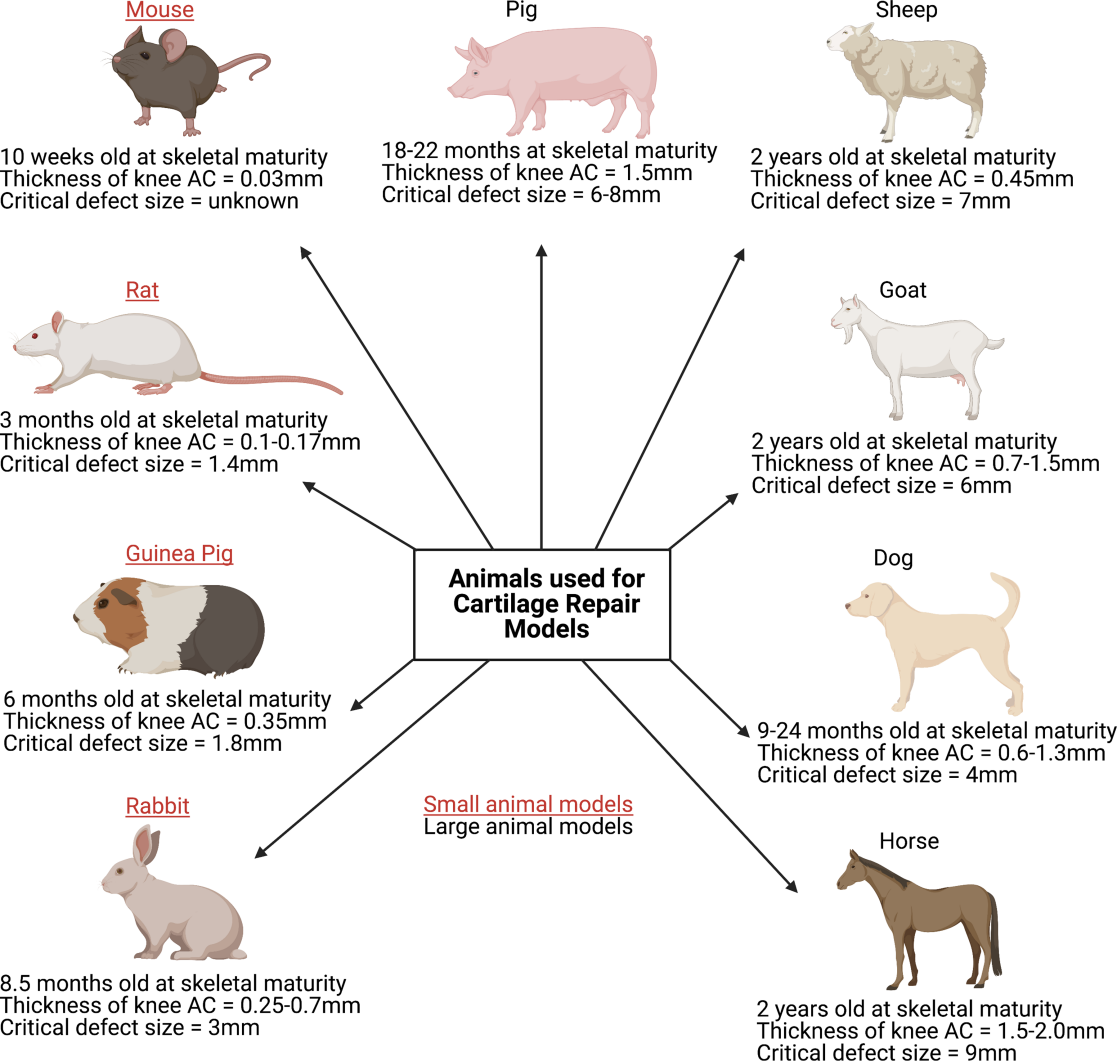
Selected characteristics of animals used for cartilage repair models. The most commonly used small animal models include the mouse, rat, guinea pig and rabbit, whilst typically the dog, sheep, goat, pig or horse are considered ‘large animal’ models. With numerous models currently available, choosing the most appropriate remains a challenge, although it is vital to note that a single model cannot encompass all of the extensive aspects involved in human cartilage repair [87]. Both small and large animals have their advantages and disadvantages for example; small animal models reach skeletal maturity faster, thereby reducing husbandry costs, experimental durations, drug and housing requirements. However, larger animals present with greater anatomical similarity in regards to the thickness of articular cartilage, joint size and biomechanics to humans. For example, in mice, the average articular cartilage thickness (mm) in the knee joint is ∼0.03 mm, whereas in horses it is ∼1.5–2.00 mm and in humans, it is ∼2.2–2.5 mm [87–91]. Both chondral and osteochondral defects of varying sizes are used in cartilage repair models; all known critical-size defects in the knee joint from the different animal species are displayed here [87,89,92]. (Created using Biorender.com.).

Studying the mechanisms and pathways of cartilage repair in humans remains a challenge, as there is restricted availability of diseased tissues, particularly at the early stage of OA in humans [72]. As a result of this, animal models remain an increasingly popular choice for basic science studies to identify the underlying molecular mechanisms of both cartilage degeneration and its progression to OA, cartilage repair or regeneration, as well as studying pharmacological interventions longitudinally (Figure 4). While spontaneous OA models exist (e.g. in mice, guinea pigs, rabbits and dogs) [73], surgical models of joint injury can provide numerous advantages, including reduced variability, a wider range of disease stages, reduced reliance on genotype and a faster onset of disease, thereby shortening study timeframes and husbandry costs [72,73]. However, it is difficult to validate these models and quantify their relevance to the development of OA in man, which tends to develop much slower and over many years.

Typically, in order to progress a novel therapy from bench-to-bedside, it needs to undergo safety and efficacy testing in pre-clinical models, often to fulfil the regulatory requirement for licensing by, for example, the MHRA or the Federal and Drug Administration (FDA). Both small and large animal models have been developed and used to investigate the pathogenesis of cartilage defects and OA, for example, the joint surface injury model in mice [74] and the OA induction model in sheep [75]. Choosing the most appropriate animal model depends on the following: the duration, objective and type of study (as different animals enter skeletal maturity at different ages, have different gestation periods and life-spans), outcome measures, ease of handling, treatment and maintenance costs. Furthermore, different regulatory approvals are required for different animal models and the genetics of the animals also need to be considered. Hence various models develop different types of cartilage injury to end-stage OA and have varying repair capacities, e.g. rabbits have a high intrinsic repair capacity, whereas humans have a lack of intrinsic repair [76]. It is therefore advisable for researchers focusing on pre-clinical models to consult and consider the key design queries outlined in the design and execution of protocols for animal research and treatment (DEPART), prior to experimental start-up as well as during and after the experimental procedure [77].

## Clinical trials using culture-expanded MSCs

Culture-expanded MSCs sourced from the bone marrow, adipose tissues and umbilical cords represent the second most commonly used cell type in clinical cartilage repair strategies. They have been utilised for the treatment of isolated cartilage defects, as well as in the treatment of early to moderate OA. MSCs have been in clinical use for a relatively short period of time, consequently, the available body of evidence supporting their use is significantly less compared with that for chondrocytes. Wakitani [78] was the first to report the results of high tibial osteotomy with or without BM-MSC-transplantation in 23 patients with OA. He found that in the MSC treated group, arthroscopic and histological scores were improved at around 42 weeks although clinical improvements were not significantly different between groups [78].

A systematic review of 17 studies of the application of intra-articular MSCs for cartilage repair and OA (11 with culture-expanded cells and 6 not) is fairly critical of the quality of trials and studies in this area, concluding that whilst benefit has been demonstrated in the short term in some cases, the evidence is limited and there is a need for further high-quality studies with long-term follow-up to validate the clinical efficacy of MSC therapy in knee OA [79].

A current ongoing clinical trial (http://clinicaltrials.gov: NCT03477942) is aiming to ‘evaluate the safety and tolerability of a single intra-articular injection of autologous BM-MSCs in 16 subjects, 8 who have knee OA and 8 who have a focal chondral defect in the knee’. A published study of adipose-derived MSCs injected into the knees of 24 patients with isolated cartilage lesions demonstrated improved International Knee Documentation Committee (IKDC), Tegner and MRI scores at 29 months [80]. A new clinical trial aiming to ‘confirm cartilage regeneration through arthroscopy after a single administration of autologous adipose tissue-derived mesenchymal stem cells (JOINTSTEM) in patients with degenerative arthritis’ has also very recently been registered and is due to start recruiting in July 2021 (http://clinicaltrials.gov: NCT04821102).

CARTISTEM® is an umbilical cord blood-derived MSC product combined with sodium hyaluronate which has been used in several studies and trials as a therapeutic agent for cartilage regeneration (http://clinicaltrials.gov: NCT01041001, NCT01626677, NCT01733186). In a 7-year follow-up study of seven patients receiving CARTISTEM®, visual analogue score (VAS) and the IKDC subjective scores were improved at 24 weeks and maintained for up to 7 years. Furthermore, histological assessments of the repair tissue indicated the presence of hyaline-like cartilage and MRIs at 3 years showed a persistence of regenerated cartilage [81]. A Phase III CARTISTEM® randomised multicentre trial has recently reported on 5-year outcomes from 89 patients treated with either CARTISTEM® or microfracture [82]. At 48 weeks, histological improvement was seen in 97.7% of the CARTISTEM® treated patients vs. 71.7% in the microfracture group. Improvement in VAS pain, WOMAC and IKDC scores were not significantly different between the treatment groups at 48 weeks, but were improved over baseline in the CARTISTEM® treated patients at 3–5-year follow-up whereas they had deteriorated at this time point in the microfracture patients [82].

### Chondrocytes and MSCs

There are few published studies to have directly compared the efficacy of MSCs versus the gold standard (ACI) [16] technique. A level 3 evidence cohort study compared 36 ACI v 36 expanded MSCs and showed no significant clinical differences in outcome up to 24 months, although outcomes were better in <45 yr old patients in ACI treated participants [83]. A recent level 2 cohort study compared the outcomes of 72 patients who received either ACI or BM-MSC transplantation. They reported no significant difference in any of the patient-reported outcomes measures between cohorts at 10 years mean follow-up [84].

The IMPACT trial (http://clinicaltrials.gov: NCT02037204) assessed the safety and efficacy of a single-stage procedure for focal cartilage lesions in the knee using a combination of autologous chondrocytes and allogeneic BM-MSCs. In this study, 35 patients were treated using autologous ‘chondrons’ and allogeneic MSCs in a fibrin glue carrier. The 5-year outcome data demonstrated that the majority of patients showed statistically significant and clinically relevant improvement in the KOOS and all its subscales from baseline [85]. The study protocol for a follow-on 60 patient randomised placebo-controlled IMPACT2 trial has now been outlined [86] and is currently recruiting (http://clinicaltrials.gov: NCT04236739).

An ongoing trial aiming to elucidate whether culture-expanded MSCs or chondrocytes (either alone or in combination) provides superior clinical benefit for the repair of cartilage defects is the autologous stem cells chondrocytes or the two (ASCOT) trial (https://www.isrctn.com: ISRCTN98997175), which is due to complete in 2023. The ASCOT trial, recruiting 114 patients with a 15 month follow-up, aims to evaluate directly which cell type (autologous bone marrow-derived MSCs or chondrocytes, alone or in combination) is the most beneficial to patients. A series of exploratory objectives (evaluating the properties of the cells and the environment that the cells are transplanted into) aim to elucidate the biological reasons for any clinical benefits observed [46].

## Conclusion

With the use of cell therapy for cartilage repair being one of the front-runners in the regenerative medicine field, it is perhaps now at an important crossroads. The current use of ambiguous terms to describe cell therapies, in general, is both limiting our scientific understanding of the basic attributes of cell therapies and undermining clinical practice [71]). Improved and more standardised nomenclature regarding the cells, such as the use of the DOSES tool [71], would help, as might improved characterisation, e.g. with transformative technologies, such as large scale omics or use of single-cell sequencing, of cell therapy products, whether ATMPs or minimally manipulated cells [37]. This in turn could guide regulatory control, which is currently cumbersome and a definite hurdle to entering the field, both clinically and commercially. Hopefully, these can be addressed in the not too distant future to move cell therapy in orthopaedics to its next phase, perhaps delivering well characterised, efficacious, allogeneic cell products in a simple and single procedure, e.g. via injection.

## Summary

- Cell therapy for treating chondral/osteochondral defects is an excellent exemplar of a successful regenerative medicine, which has provided meaningful clinical improvement in patients for nearly three decades.
- Whilst autologous chondrocyte implantation has been shown to be cost-effective, its widespread use has been restricted principally by the governance and licensing associated with an autologous cell therapy.
- A clinically efficacious allogeneic cell therapy, delivered in a minimally invasive manner, has the potential to result in a paradigm shift in cell therapy for chondral defects. An allogeneic therapy may reduce current regulatory barriers to use in the clinic and should reduce manufacturing costs, improving its cost-effectiveness.
- However, continuing improvements in transformative scientific technologies, will help us better understand the main mechanism of action of MSCs, whether that be in influencing the environment in which they are transplanted or whether the cells themselves differentiate into chondrocytes.

## Abbreviations

ACI: autologous chondrocyte implantation
ACTIVE: autologous chondrocyte transplantation/implantation versus existing treatments
ASCOT: autologous stem cells chondrocytes or the two
ATMP: advanced therapeutic medicinal product
BMA: bone-marrow aspirate
BMAC: bone-marrow aspirate concentrate
GMP: good manufacturing practice
IKDC: International Knee Documentation Committee
KOOS: Knee injury and Osteoarthritis Outcome Score
MSCs: mesenchymal stromal cells
OA: osteoarthritis
OATS: osteochondral autograft transplantation
VAS: visual analogue score
eSCs: embryonic stem cells
